# Increase of the Adiponectin/Leptin Ratio in Patients with Obesity and Type 2 Diabetes after Roux-en-Y Gastric Bypass

**DOI:** 10.3390/nu11092069

**Published:** 2019-09-03

**Authors:** Xabier Unamuno, Maitane Izaguirre, Javier Gómez-Ambrosi, Amaia Rodríguez, Beatriz Ramírez, Sara Becerril, Víctor Valentí, Rafael Moncada, Camilo Silva, Javier Salvador, Piero Portincasa, Gema Frühbeck, Victoria Catalán

**Affiliations:** 1 Metabolic Research Laboratory, Clínica Universidad de Navarra, 31008 Pamplona, Spain; 2 CIBER Fisiopatología de la Obesidad y Nutrición (CIBEROBN), Instituto de Salud Carlos III, 31008 Pamplona, Spain; 3 Obesity and Adipobiology Group, Instituto de Investigación Sanitaria de Navarra (IdiSNA), 31008 Pamplona, Spain; 4 Department of Surgery, Clínica Universidad de Navarra, 31008 Pamplona, Spain; 5 Department of Anesthesia, Clínica Universidad de Navarra, 31008 Pamplona, Spain; 6 Department of Endocrinology & Nutrition, Clínica Universidad de Navarra, 31008 Pamplona, Spain; 7 Clinica Medica “A. Murri”, Department of Biomedical Sciences and Human Oncology, University of Bari “Aldo Moro” Medical School, 70124 Bari, Italy

**Keywords:** adiponectin, leptin, adiponectin/leptin ratio, obesity, type 2 diabetes, weight loss, Roux-en-Y gastric bypass

## Abstract

Bariatric surgery remains the most effective option for achieving important and sustained weight loss. We explored the effects of Roux-en-Y gastric bypass (RYGB) on the circulating levels of adiponectin, leptin, and the adiponectin/leptin (Adpn/Lep) ratio in patients with obesity and type 2 diabetes (T2D). Twenty-five T2D volunteers undergoing RYGB were included in the study, and further subclassified as patients that responded or not to RYBG, regarding remission of T2D. Anthropometric and biochemical variables were evaluated before and after RYGB. Obese patients with T2D exhibited an increase (*p* < 0.0001) in the Adpn/Lep ratio after RYGB. Changes in the Adpn/Lep ratio correlated better with changes in anthropometric data (*p* < 0.001) than with the variations of adiponectin or leptin alone. Multiple regression analysis revealed that the change in the Adpn/Lep ratio in patients with T2D was an independent predictor of the changes in body mass index (*p* < 0.001) and body fat percentage (*p* = 0.022). However, the Adpn/Lep ratio did not differ between individuals with or without T2D remission after RYGB. In summary, the current study demonstrated that after weight and body fat loss following RYGB, the Adpn/Lep ratio increased in patients with obesity and T2D.

## 1. Introduction

The prevalence of overweight and obesity is increasing worldwide, reaching epidemic proportions and emerging as a major public health challenge [1,2]. Trends are alarming because obesity affects the function of different organ systems, shortening life span, and conferring an increased risk for multiple serious conditions, including type 2 diabetes (T2D), cardiovascular diseases, non-alcoholic fatty liver disease, and different types of cancers [3]. The management of obesity is often difficult or unsuccessful due to its multifactorial nature [4]. Treatment options involve lifestyle interventions (caloric restriction, reduction of sedentary behaviors, and increased physical activity), pharmacotherapy as well as surgical procedures [5,6]. Conventional treatments have little effect on weight loss and are relatively inefficient in treating obesity in the long-term [7,8,9]. By contrast, bariatric surgery has rapidly expanded, due to its capacity to induce sustained long-term weight loss, ameliorate obesity-related comorbidities, and reduce mortality, constituting an effective option for individuals with severe obesity [6,10].

Even though bariatric surgery leads to the improvement of T2D, differences related to the resolution of the obesity-related conditions exist [11]. Therefore, the identification of prediction factors of T2D remission after bariatric surgery is an important topic [12,13]. Factors such as the preoperative duration of T2D, the use of insulin, as well as a high percentage of glycated hemoglobin (HbA1c) and lower weight loss after surgery can influence the remission of T2D [14,15]. Among these factors, different studies have described the potential role of adipokines in weight loss and T2D remission after surgery [16,17,18]. Adiponectin has strong anti-inflammatory and insulin-sensitizing functions and their plasma levels have been clearly demonstrated to be decreased as the body mass index (BMI) increases being oppositely correlated with insulin resistance [19,20]. Increased adiponectin levels after bariatric surgery are associated with metabolic benefits and with a higher rate of T2D remission [21,22]. By contrast leptin, another adipocyte-derived factor, parallels the degree of adiposity and is associated with insulin resistance [23,24]. After bariatric surgery, leptin concentrations decrease in proportion to post-operatively achieved weight loss [25]. The balance between these adipose tissue-derived hormones has a pivotal role in evaluating the metabolic outcome of bariatric surgery. We proposed the adiponectin/leptin (Adpn/Lep) ratio as a marker of adipose tissue dysfunction and inflammation [26,27], being better correlated with insulin resistance than adiponectin or leptin alone [28]. Furthermore, we found a decreased Adpn/Lep ratio in patients with the metabolic syndrome [28] and a gradual reduction with increasing number of risk factors for metabolic syndrome has been also reported [29,30]. Patients with obesity and type 1 diabetes (T1D) also exhibited a significantly lower Adpn/Lep ratio compared to non-obese T1D patients [31]. Moreover, the Adpn/Lep ratio has shown to estimate insulin sensitivity [32,33] and the risk of cardiovascular diseases in patients with the acquired immune deficiency syndrome [34,35]. In this regard, new cutoffs to assess metabolic risk based on the Adpn/Lep ratio have been proposed [26]. An Adpn/Lep ratio higher than 1.0 can be considered as normal whereas a ratio below 0.5 may indicate an increase in the metabolic risk (with adiponectin concentrations measured in μg/mL and leptin levels in ng/mL) [26].

Since bariatric surgery induces sustained weight and fat loss and improves the resolution of T2D together with an increase of adiponectin concentrations and a reduction in leptin levels, we aim to investigate the Adpn/Lep ratio in patients with T2D before and after weight loss achieved by bariatric surgery. In addition, we analyzed whether the Adpn/Lep ratio varies among groups of patients with different outcomes of T2D improvement after Roux-en-Y gastric bypass (RYGB).

## 2. Materials and Methods

### 2.1. Patient Selection

A group of 25 patients with obesity and T2D (11 males and 14 females) recruited from patients visiting the Departments of Endocrinology & Nutrition and Surgery for weight loss treatment at the Clínica Universidad de Navarra was used to analyze the effect of RYGB on the Adpn/Lep ratio one year after surgery. The clinical assessment was performed by a multidisciplinary consultation team. Individuals with severe systemic disease not related to obesity, infectious disease, cancer, liver disease or severe nephropathy, pregnancy or lactation, patients with serious eating disorders, and people whose freedom is under legal or administrative requirement were excluded. The experimental design was approved by the Research Ethics Committee of the University of Navarra (protocol 2017.126) and the study followed the ethical standards of the Declaration of Helsinki and its later amendments. All volunteers signed the informed consent to participate in the study.

Obese patients were diagnosed as patients with T2D following the criteria of the Expert Committee on the Diagnosis and Classification of Diabetes [36]. In order to be able to identify potential differences among patients as regards T2D remission, volunteers were studied one year after RYGB. To make sure that the improvement in their glucose metabolism was consistent, the classification of the same patients as responders or non-responders was made upon the analytical values obtained 3 years after RYGB. The volunteers maintained the classification after the 3 years. Remission of T2D in obese patients was defined according to the American Diabetes Association criteria [36]. Specifically, remission was defined by HbA1c < 6.0%, fasting glucose < 100 mg/dL, and no use of antidiabetic medication for at least 12 months. The novel scoring system (DiaRem score) to estimate the probability of T2D remission after RYGB was also calculated based on age, preoperative HbA1c, as well as the use of metformin, sulfonylurea, glitazones, and/or insulin, as previously reported [12]. BMI was calculated as weight in kilograms (kg) divided by the height in meters squared and body fat (BF) was estimated by air-displacement-plethysmography (Bod-Pod^®^, Life Measurements, Concord, CA, USA) as previously described [37]. The waist-to-hip ratio (WHR) was calculated as the ratio of waist circumference (at the midpoint between the margin of the last palpable rib and the top of the iliac crest) to hip circumference (at the widest trochanters).

### 2.2. Analytical Procedures

Plasma samples were obtained by venipuncture after an overnight fast. Glucose was analyzed by an automated analyzer (Roche Cobas 8000, Roche, Basel, Switzerland). Insulin was measured by an automated enzyme immunoassay (IMMULITE^®^ 2000 XPi, Siemens, Malvern, PA, USA) with intra- and inter-assay coefficients of variation of 4.2% and 5.7%, respectively. Insulin sensitivity and resistance were calculated using the quantitative insulin sensitivity check index (QUICKI) and homeostasis model assessment (HOMA) indices, respectively. Fasting C-peptide levels and 6-min after an intravenous injection of 1 mg of glucagon were measured by an automated enzyme immunoassay (IMMULITE^®^ 2000 XPi, Siemens). Total cholesterol and triglyceride concentrations were determined by enzymatic spectrophotometric methods (Boehringer Mannheim, Mannheim, Germany). High-density lipoprotein (HDL-cholesterol) was quantified by a colorimetric method in a Beckman Synchron^®^ CX analyzer (Beckman Instruments, Ltd., Bucks, UK) and low-density lipoprotein (LDL-cholesterol) was calculated using the Friedewald formula as previously described [27]. Uric acid, creatinine, and the hepatic enzymes alanine aminotransferase (ALT), aspartate aminotransferase (AST), and γ-glutamyltransferase (γ-GT) were analyzed in an automated analyzer (Roche/Hitachi Modular P800). Fibrinogen, high-sensitivity C-reactive protein (CRP) and von Willebrand factor antigen (vWF) levels were measured as previously reported [27]. Leptin levels were quantified by a double-antibody radioimmunoassay method (Linco Research, Inc., St. Charles., MO, USA) as previously described [38]. The intra- and inter-assay coefficients of variation were 5.0% and 4.5%, respectively. Adiponectin was determined by a commercially available ELISA kit (Biovendor, Brno, Czech Republic) with intra- and inter-assay coefficients of variation being 3.9% for the former and 4.2% for the latter. The Adpn/Lep ratio was calculated with adiponectin concentrations expressed in μg/mL and leptin levels in ng/mL [26].

### 2.3. Statistical Analysis

Data are presented as mean ± standard error of the mean (SEM). Differences in the proportion of subjects within groups regarding gender were assessed by the Chi-square test. Differences between groups were assessed by two-tailed paired or unpaired Student’s *t* tests as appropriate. Correlations between two variables were computed by Pearson’s correlation coefficient (r). The calculations were performed using the SPSS/Windows version 15.0 statistical package (SPSS, Chicago, IL, USA). A *p* value < 0.05 was considered statistically significant.

## 3. Results

Anthropometric and biochemical characteristics of the subjects enrolled in the study are summarized in Table 1. All the subjects included in the study were classified as patients with obesity and T2D. From the whole cohort, 44% were males and 56% were females, with no differences in gender distribution (*p* = 0.549). As expected, after an average post-surgical period of one year, patients experienced a significant decrease (*p* < 0.0001) in all anthropometric measurements (BMI, body adiposity, waist circumference, and WHR). Furthermore, insulin resistance improved as evidenced by the decrease in fasting glucose (*p* < 0.05) and insulin concentrations (*p* < 0.001), together with a decrease (*p* < 0.001) in the HOMA as well as an increase (*p* < 0.001) in the QUICKI indices. After the first postoperative year, the HbA1c mean values decreased significantly (*p* < 0.01). Volunteers also had improved lipid metabolism supported by a significant reduction (*p* < 0.01) in circulating triglycerides, total- and LDL-cholesterol as well as an increase (*p* < 0.01) in HDL-cholesterol concentrations. Noteworthy, a decrease in the circulating concentrations of the inflammatory markers CRP (*p* < 0.05) and uric acid (*p* < 0.001), as well as in the levels of γ-GT (*p* < 0.01), a marker of hepatobiliary injury, after weight loss, were detected.

**Figure 1:**
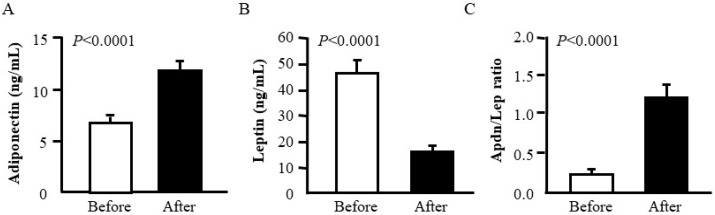
Comparison of serum adiponectin (**A**) and leptin (**B**) concentrations as well as the Adiponectin/Leptin (Adpn/Lep) ratio (**C**) before and after Roux-en Y gastric bypass in patients with obesity and T2D (*n* = 25). Statistical differences were analyzed by two-tailed paired Student’s *t* test.

RYGB was associated with an increase (*p* < 0.0001) in adiponectin together with a decrease (*p* < 0.0001) in leptin concentrations. Importantly, the Adpn/Lep ratio increased significantly after weight loss (from 0.21 ± 0.03 to 1.20 ± 0.19; *p* < 0.0001) (Figure 1). According to the cutoff points defining cardiometabolic risk previously proposed by our group [26], following bariatric surgery, patients with T2D showed an increase in the Adpn/Lep ratio from below 0.5 (indicative of a severe increase in their cardiometabolic risk) to over 1.0 (considered as normal).

We also detected a highly positive correlation between post-operative values of the Adpn/Lep ratio and changes in anthropometric characteristics (Table 2). To further strengthen the reliability of the Adpn/Lep ratio as an important marker of weight and body fat loss after RYBG, we compared the degree of correlation between changes in the Adpn/Lep ratio and the variations in body composition-related variables as opposed to each adipokine separately (Table 3). Interestingly, changes in Adpn/Lep ratio provided better correlations with differences in BMI (r = −0.64, *p* < 0.001), body fat (r = −0.80, *p* < 0.001), waist circumference (r = −0.76, *p* < 0.001), and WHR (r = −0.52, *p* = 0.011), compared with the associations of the leptin and adiponectin concentration changes alone.

We next addressed whether the Adpn/Lep ratio differed between patients with T2D that responded or not to RYGB regarding remission of T2D. For both groups of T2D obese patients, follow-up data beyond the third postoperative year were available (Table 4). Overall, we categorized the 25 patients with T2D who underwent RYGB surgery as non-responders (*n* = 7, 28%) and responders (*n* = 18, 72%). As expected, non-responders showed a significantly higher DiaRem score compared to those classified as responders (10.30 ± 1.72 vs. 4.27 ± 0.86; *p* = 0.008). Although fasting C-peptide levels were higher in responders (4.22 ± 0.27 ng/mL) compared to non-responders (3.21 ± 0.69 ng/mL), differences did not reach statistical significance. C-peptide levels 6-min after intravenous injection of 1 mg of glucagon followed the same trend, being increased in patients with T2D remission (responders: 6.79 ± 0.56 ng/mL vs. non-responders: 5.86 ± 0.46 ng/mL). However, differences were not statistically significant. Both groups of patients exhibited an increased Adpn/Lep ratio but no significant differences between individuals who experienced a T2D remission or not after RYGB were found.

## 4. Discussion

A huge body of knowledge supports the surgical management as the most effective treatment for severe obesity, being associated with a significant reduction of mortality [6]. The Adpn/Lep ratio might represent a useful biomarker of dysfunctional adipose tissue because it is negatively correlated with markers of low-grade chronic inflammation [26,27,28]. In this regard, we designed the present study to determine changes in the Adpn/Lep ratio after RYGB in obese patients with T2D. Indeed, obese patients with T2D exhibited an increase in the Adpn/Lep ratio after RYGB. Consistently, we also found that changes in the Adpn/Lep ratio correlated better with changes in anthropometric data than the variations of adiponectin or leptin alone. However, there was no difference in the Adpn/Lep ratio between individuals who experienced a remission or not of T2D after RYGB.

Adipose tissue constitutes an active and potent endocrine organ capable of releasing a large number of adipokines involved in the pathophysiological link between increased adiposity and its associated metabolic alterations [23]. Among these factors, important studies have shown increased and decreased circulating levels of leptin and adiponectin, respectively, in the obese state as well as their significant role in the regulation of weight loss and glycaemic control [23,39,40]. The usefulness of the combination of both adipocyte-derived hormones in the Adpn/Lep ratio has been clearly demonstrated, concluding that it could be a better marker for insulin resistance rather than these adipokines individually [26,27,33,41,42]. In this sense, we have recently described that a low Adpn/Lep ratio is associated with a dysfunctional adipose tissue [26,27]. In this study, subjects with obesity and T2D showed an increase in the Adpn/Lep ratio after RYGB. Reportedly, low-to-moderate weight loss in adolescents after conventional treatment (diet, physical exercise, and clinical support) promoted a significant increase in the Adpn/Lep ratio, with changes in lean body mass constituting an independent predictor of changes in this ratio [43]. Moreover, the Mediterranean diet led to increased Adpn/Lep ratio, mainly associated to its efficacy in reducing abdominal fat [41].

The reduction in anthropometric measurements, specifically in visceral fat, after RYGB may underlie the increase of the Adpn/Lep ratio in our patients with T2D, being associated with important and positive health consequences. Consequently, we also determined that changes in the Adpn/Lep ratio were better associated with changes in BMI, body fat, and waist circumference than the changes in individual levels of leptin and adiponectin. The Adpn/Lep ratio has been also correlated with insulin resistance more closely than both hormones alone or even the HOMA index [33]. These results reinforce the importance of RYGB for the treatment of obesity targeting a reduction of body fat, mainly the visceral depot. Furthermore, the Adpn/Lep ratio better predicts the risk of cardiovascular diseases, as compared to the individual leptin and adiponectin concentrations [44,45]. We recently found that a low Adpn/Lep ratio is associated with an increased cardiometabolic risk, evidenced by elevated systemic inflammation and oxidative stress [28]. In addition, the Adpn/Lep ratio negatively correlates with the markers of inflammation SAA and CRP, suggesting that this marker may reflect the systemic inflammation in the context of obesity [27,46].

No differences in the Adpn/Lep ratio at one year between individuals who experienced a T2D remission or not three years after RYGB existed in our study. Of note, age is a common denominator of diabetes remission across multiple studies, despite different surgical types and remission criteria [12,47]. In this sense, patients from this study, not experiencing a remission in T2D, were older and exhibited a deteriorated pancreatic β-cell function with insulinopenia. This finding is being probably related to their impaired diabetes remission after bariatric surgery. C-peptide is considered an important determinant of T2D remission since it constitutes a direct measure of insulin production and provides an accurate measure of pancreatic β-cell reserve [48,49]. Higher C-peptide levels have been described in subjects with T2D remission compared to those without remission suggesting that preoperative C-peptide determination may be a useful estimator of T2D remission after bariatric surgery [50]. Although differences were not statistically significant, we found that fasting C-peptide and C-peptide levels 6-min after intravenous injection of 1 mg of glucagon were higher in responders compared to non-responders. Patients without diabetes remission at three years after RYGB included in the study suffered from NAFLD, which could explain the higher ratio of the Adpn/Lep ratio without T2D remission. Further studies with a larger number of patients would clarify the involvement of the Adpn/Lep ratio in the prediction of T2D remission after bariatric surgery. The potential impact of other factors known to be changed following bariatric surgery like fibroblast growth factors, caveolin-1, or aquaporins [51,52,53] as well as their plausible influence on relevant aspects of adipobiology such as lipolysis [54,55] should not be discarded.

## 5. Conclusions

In conclusion, we showed that the increase in the Adpn/Lep ratio after RYGB might constitute a significant factor for the body composition and metabolic benefits after bariatric procedures. The ratio, however, does not represent a predictive factor for the remission of T2D. Due to the complex regulation of hormonal and body composition alteration involved in weight loss, the balance between the anti-inflammatory adipokine, adiponectin, and its inflammatory counterpart, leptin, may be of great importance in determining the postoperative weight and fat loss as well as the metabolic outcome of bariatric surgery. Further prospective studies will assess the involvement of the Adpn/Lep ratio after bariatric surgery in the prevention of serious obesity-related complications.

## Figures and Tables

**Table 1:** Anthropometric and metabolic effects in patients with obesity and type 2 diabetes (T2D) before and after Roux-en-Y gastric bypass (RYGB).

Characteristics	Before Surgery	After Surgery
*n* (males, females)	25 (11, 14)	25 (11, 14)
Age (years)	50 ± 2	51 ± 2
BMI (kg/m^2^)	44.2 ± 1.3	33.6 ± 1.6 ***
Body fat (%)	49.9 ± 1.5	39.6 ± 2.0 ***
Waist (cm)	128 ± 3	107 ± 3 ***
Waist-to-hip ratio	1.00 ± 0.01	0.97 ± 0.02 **
SBP (mmHg)	128 ± 3	120 ± 3 **
DBP (mmHg)	80 ± 2	73 ± 1 ***
Fasting glucose (mg/dL)	133 ± 7	115 ± 9
Fasting insulin (μU/mL)	21.3 ± 2.5	8.0 ± 1.2 ***
HOMA	6.6 ± 0.8	2.1 ± 0.3 ***
QUICKI	0.302 ± 0.006	0.363 ± 0.010 ***
HbA1c (%)	7.2 ± 0.2	6.4 ± 0.2 **
Triglycerides (mg/dL)	140 ± 10	97 ± 11 **
Total cholesterol (mg/dL)	185 ± 7	159 ± 7 *
LDL-cholesterol (mg/dL)	111 ± 6	90 ± 5 *
HDL-cholesterol (mg/dL)	46 ± 2	51 ± 2 *
Leptin (ng/mL)	45.3 ± 5.6	15.1 ± 2.4 ***
Adiponectin (µg/mL)	6.73 ± 0.67	11.68 ± 0.81 ***
Adpn/Lep ratio	0.21 ± 0.03	1.20 ± 0.19 ***
Uric acid (mg/dL)	5.8 ± 0.3	4.5 ± 0.2 ***
Creatinine (mg/dL)	0.80 ± 0.04	0.77 ± 0.03 *
CRP (mg/L)	8.5 ± 1.6	2.1 ± 1.0 *
Fibrinogen (mg/dL)	385 ± 21	348 ± 23
von Willebrand factor (%)	152 ± 11	138 ± 14
Homocysteine (μmol/L)	10.2 ± 1.2	9.3 ± 1.1
AST (IU/L)	15 ± 1	18 ± 2
ALT (IU/L)	21 ± 2	27 ± 5
AST/ALT	0.78 ± 0.07	0.87± 0.07
γ-GT (IU/L)	35 ± 6	16 ± 2 **

Adpn/Lep, adiponectin/leptin ratio; ALT, alanine aminotransferase; AST, aspartate aminotransferase; BMI, body mass index; CRP, C-reactive protein; DBP, diastolic blood pressure; γ-GT, γ-glutamyltransferase; HbA1c, glycated hemoglobin; HOMA, homeostasis model assessment; NG, normoglycemic; QUICKI, quantitative insulin sensitivity check index; SBP, systolic blood pressure; T2D, type 2 diabetes. Data are mean ± SEM. Differences between groups were analyzed by two-paired Student *t* tests. * *p* < 0.05, ** *p* < 0.01, and *** *p* < 0.001 vs. before surgery.

**Table 2:** Univariate analysis of the correlations between the adiponectin/leptin ratio before and after Roux-en-Y gastric bypass (RYGB) with changes in anthropometric characteristics.

	Adpn/Lep Ratio Before Surgery		Adpn/Lep Ratio After Surgery	
	r	*p*	r	*p*
Δ BMI	−0.36	0.077	0.58	0.002
Δ BF	−0.02	0.912	0.79	<0.001
Δ Waist	−0.14	0.526	0.72	<0.001
Δ WHR	0.37	0.087	0.58	0.004

Adpn/Lep ratio, adiponectin/leptin ratio; BMI, body mass index; BF, body fat; WHR, waist-to-hip ratio; r, Pearson correlation coefficient; *p*, significance value.

**Table 3:** Univariate analysis of the correlations between the differences in adiponectin and leptin concentrations as well as in the adiponectin/leptin ratio with changes in anthropometric characteristics after Roux-en-Y gastric bypass (RYGB).

	Δ Adiponectin		Δ Leptin		Δ Adpn/Lep Ratio	
	r	*p*	r	*p*	r	*p*
Δ BMI	−0.49	0.013	0.73	<0.001	−0.64	<0.001
Δ BF	−0.48	0.014	0.25	0.234	−0.80	<0.001
Δ Waist	−0.45	0.030	0.58	0.004	−0.76	<0.001
Δ WHR	−0.06	0.788	0.06	0.783	−0.52	0.011

Adpn/Lep ratio, adiponectin/leptin ratio; BMI, body mass index; BF, body fat; WHR, waist-to-hip ratio; r, Pearson correlation coefficient; *p*, significance value.

**Table 4:** Effects of weight loss one year after Roux-en-Y gastric bypass (RYGB) in obese patients with T2D classified as responders and non-responders.

	Responders		Non-Responders		*p* (R vs. NR)	
	Before BS	After BS	Before BS	After BS	Before BS	After BS
*n*	18	18	7	7	-	-
Age	49 ± 3	50 ± 3	58 ± 2	59 ± 2	0.014	0.014
BMI (kg/m^2^)	45.2 ± 1.7	33.8 ± 1.6 ***	41.4 ± 1.9	32.3 ± 2.6 **	0.226	0.601
Body fat (%)	50.7 ± 1.9	40.2 ± 2.5 ***	49.2 ± 2.9	38.4 ± 4.1 *	0.273	0.701
Waist (cm)	127 ± 4	106 ± 4 ***	105 ± 4	110 ± 6 **	0.731	0.730
Waist-to-hip ratio	0.99 ± 0.01	0.96 ± 0.02	1.02 ± 0.02	0.95 ± 0.02 *	0.246	0.836
SBP (mmHg)	127 ± 4	117 ± 3 *	130 ± 4	126 ± 7 **	0.679	0.220
DBP (mmHg)	81 ± 3	72 ± 2 ***	77 ± 4	74 ± 3 *	0.439	0.628
Fasting glucose (mg/dL)	125 ± 6	97 ± 4 ***	166 ± 16	172 ± 25	0.004	0.002
Fasting insulin (μU/mL)	21.9 ± 3.2	9.5 ± 1.5 **	19.1 ± 4.9	4.7 ± 1.0 *	0.649	0.008
HOMA	6.3 ± 1.0	2.3 ± 1.4 **	7.8 ± 2.2	1.8 ± 0.5 **	0.487	0.516
QUICKI	0.304 ± 0.007	0.358 ± 0.012 ***	0.298 ± 0.017	0.362 ± 0.020 *	0.728	0.869
HbA1c (%)	7.3 ± 0.6	6.1 ± 0.4 **	8.1 ± 0.5	6.7 ± 0.2	0.005	0.172
Triglycerides (mg/dL)	141 ± 12	84 ± 10 ***	129 ± 28	135 ± 30	0.422	0.041
Total cholesterol (mg/dL)	194 ± 10	150 ± 7 ***	162 ± 8	184 ± 9	0.222	0.025
LDL-cholesterol (mg/dL)	122 ± 8	86 ± 7 **	90 ± 9	100 ± 6	0.190	0.263
HDL-cholesterol (mg/dL)	45 ± 3	48 ± 2	45 ± 2	57 ± 4 *	0.513	0.058
Leptin (ng/mL)	48.8 ± 6.9	16.9 ± 3.1 ***	38.1 ± 10.8	11.8 ± 3.1 *	0.415	0.830
Adiponectin (µg/mL)	6.49 ± 0.90	11.80 ± 1.06 ***	7.34 ± 0.64	11.39 ± 1.14	0.582	0.356
Adpn/Lep ratio	0.17 ± 0.03	1.11 ± 0.21 ***	0.32 ± 0.09	1.43 ± 0.44 *	0.150	0.478
Uric acid (mg/dL)	5.9 ± 0.4	4.4 ± 0.3 **	5.3 ± 0.3	4.6 ± 0.3	0.325	0.759
Creatinine (mg/dL)	0.78 ± 0.04	1.29 ± 0.54	0.80 ± 0.09	0.74 ± 0.07 *	0.551	0.811
CRP (mg/L)	9.7 ± 4.2	3.0 ± 1.6	7.3 ± 3.1	0.9 ± 0.2 *	0.447	0.352
Fibrinogen (mg/dL)	378 ± 24	373 ± 24	357 ± 72	271 ± 32	0.828	0.154
von Willebrand factor (%)	159 ± 23	140 ± 18	161 ± 28	136 ± 27 *	0.940	0.971
Homocysteine (μmol/L)	7.92 ± 1.67	9.47 ± 1.44	11.30 ± 1.70	9.13 ± 1.11	0.722	0.838
AST (IU/L)	16 ± 2	17 ± 2	18 ± 1	31 ± 3	0.913	0.262
ALT (IU/L)	23 ± 2	26 ± 5	23 ± 3	26 ± 12	0.273	0.656
AST/ALT	0.70 ± 0.04	0.79 ± 0.06	1.00 ± 0.18	1.07 ± 0.21	0.135	0.240
γ-GT (IU/L)	32 ± 5	15 ± 2 **	43 ± 18	24 ± 7	0.439	0.233

Adpn/Lep, adiponectin/leptin ratio; ALT, alanine aminotransferase; AST, aspartate aminotransferase; BMI, body mass index; BS, bariatric surgery; CRP, C-reactive protein; DBP, diastolic blood pressure; γ-GT, γ-glutamyltransferase; HbA1c, glycated hemoglobin; HOMA, homeostasis model assessment; NG, normoglycemic; QUICKI, quantitative insulin sensitivity check index; SBP, systolic blood pressure; T2D, type 2 diabetes. Data are mean ± SEM. Differences between groups were analyzed by two-paired Student *t* tests. * *p* < 0.05, ** *p* < 0.01, and ** *p* < 0.001 vs. before surgery.
